# Gastric Angiomyolipoma, a Very Rare Cause of Upper Gastrointestinal Bleeding: A Case Report and a Brief Review of Literature

**DOI:** 10.4021/gr486e

**Published:** 2012-09-20

**Authors:** Raghava Reddy Levaka Veera, Swapna Reddy Bemalgi

**Affiliations:** a Department of Internal Medicine, Abington Memorial Hospital, Abington, PA, USA

**Keywords:** Angiomyolipoma, Gastric, GAML, HMB-45, EUS

## Abstract

Angiomyolipoma (AML) is a tumor composed of variable proportions of adipose tissue, spindle and epithelioid smooth muscle-like cells and abnormal thick-walled blood vessels which are usually benign. AML is relatively common in kidney and liver. Gastric angiomyolipoma (GAML) is extremely rare and only 2 cases were reported in the literature. Diagnosis of AML is difficult owing to its rarity and varied immunohistochemical patterns. Here, we report a case of GAML in a 65-year-old male who presented with an episode of hematemesis and intermittent melena for one week. Endoscopy showed a partially obstructing mass arising from gastric antrum with central ulceration. Mass was removed by wedge resection after laparoscopic anterior gastrectomy. Excised specimen showed a benign 6 × 3 × 3 cm homogenous fatty mass with adipose tissue, smooth muscles and prominent vascularity. Immunohistochemistry stains were positive for desmin, smooth muscle actin (SMA), CD34 and negative for human melanoma black (HMB)-45 antigen and CD117. This case reports the largest and HMB-45 negative GAML so far, which can be a very rare cause of upper gastrointestinal bleeding.

## Introduction

Angiomyolipoma (AML) is a relatively rare neoplasm, composed of variable proportions of adipose tissue, spindle and epithelioid smooth muscle-like cells and abnormal thick-walled blood vessels [1]. Though AMLs are considered to be benign tumors, there are sporadic cases of either recurrent or malignant AMLs reported in the literature [2]. The two most commonly affected organs are kidney and liver accounting > 90% of the cases [3]. In the gastrointestinal tract, other than in liver, it was reported in duodenum, appendix, pancreas and stomach [3-5]. Gastric angiomyolipoma (GAML) is extremely rare and was reported only twice in the literature [4, 5]. Here, we report the third case of GAML with typical histopathological features.

## Case Report

A 65-year-old male presented with an episode of hematemesis and intermittent melena for one week. He reported some stomach upset with decreased appetite, dizziness, and exertional shortness of breath lately. As per his wife, pt also had an episode of syncope, the day before admission. He was diagnosed with diverticulitis a week before, when he presented with fevers, chills, left lower abdominal pain, and was started on oral cephalexin and metronidazole, which he was on day 6 of the antibiotics at the time of presentation. He reported increased use of ibuprofen since he had the diverticulitis, using up to four tablets of 200 mg daily. He denied having any fevers, chills, weight loss or other sites of bleeding. His past medical history included hypertension, hyperlipidemia, cholelithiasis and Abdominal Aortic Aneurysm (AAA). Past surgical history included bilateral visceral hernia repair and vasectomy. There was no history of heart burn or reflux disease. He was a previous smoker with fifty plus pack year smoking history and quit smoking 10yrs ago, consumed alcohol socially. He denied recreational drug use. Family history included coronary artery disease on both sides of the family. He was only on simvastatin at home and did not have allergies to any medications.

**Figure 1:**
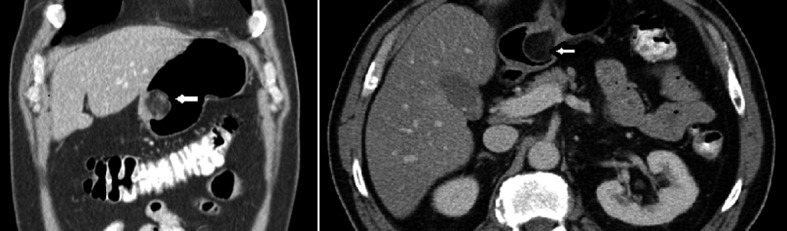
Axial and sagittal view of CT scan abdomen and pelvis showing a polypoid mass (white arrows) arising from gastric antrum.

**Figure 2:**
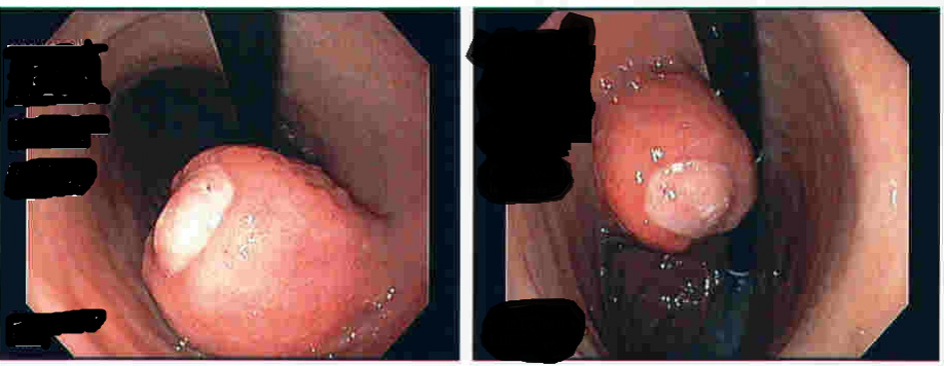
EGD showing polypoid mass with central ulceration.

In the emergency room, he was hemodynamically stable. Nasogastric lavage showed some coffee ground emesis. On physical exam, he was well oriented, chest and heart examination was normal. Abdominal examination showed mildly hyperactive bowel sounds, otherwise normal. No organomegaly was noted. Heme occult was positive. Admission lab work showed hemoglobin of 12.4 mg/dL with hematocrit of 38. Complete metabolic panel was normal. Patient was given nothing by mouth, started on intravenous fluids and protein pump inhibitor (PPI) drip. It was thought to be non-steroidal anti-inflammatory drug (NSAID) related gastropathy or peptic ulcer related bleeding and ibuprofen was stopped. Overnight, he had couple of episodes of melena resulting in hemoglobin drop and needing blood transfusion. Computerized tomography (CT) scan of abdomen/pelvis with contrast that was taken at other hospital for diverticulitis incidentally showed a gastric mass, gall stones, stable AAA (Fig. 1). No other masses were noted in the abdomen or pelvis. Esophagogastroduodenoscopy (EGD) showed a partially obstructing, polypoid mass with central ulceration arising from gastric body which might be the cause of bleeding (Fig. 2). Mild antral gastritis with no active bleeding was noted. Biopsies were taken. The differential diagnosis at that time was lipoma, gastric carcinoma versus gastrointestinal stromal tumor (GIST). Pantoprazole drip was stopped and changed to oral Pantoprazole twice daily.

**Figure 3:**
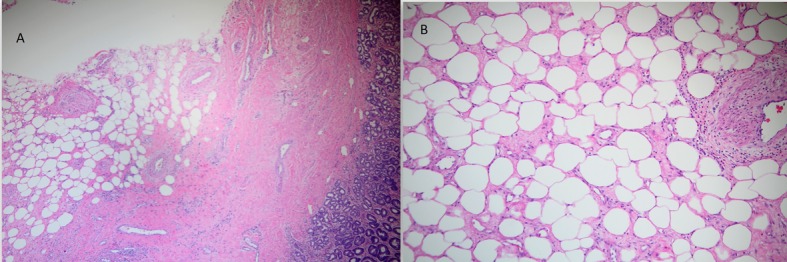
H&E staining of histopathological specimen showing varying proportions of adipose tissue, smooth muscle and blood vessels (A); magnified view (B).

**Figure 4:**
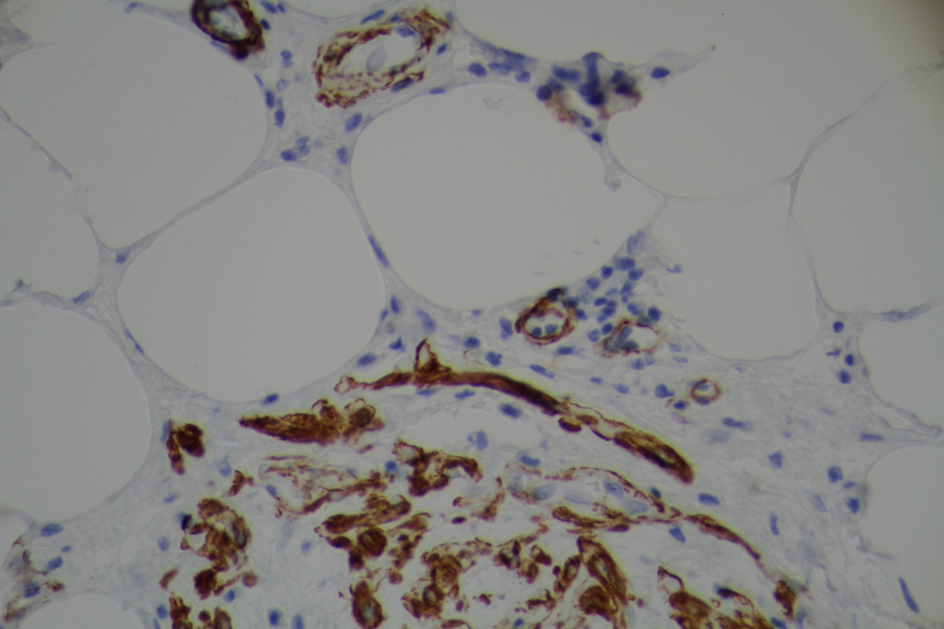
Tumor positive for immunohistochemical stain for desmin.

**Figure 5:**
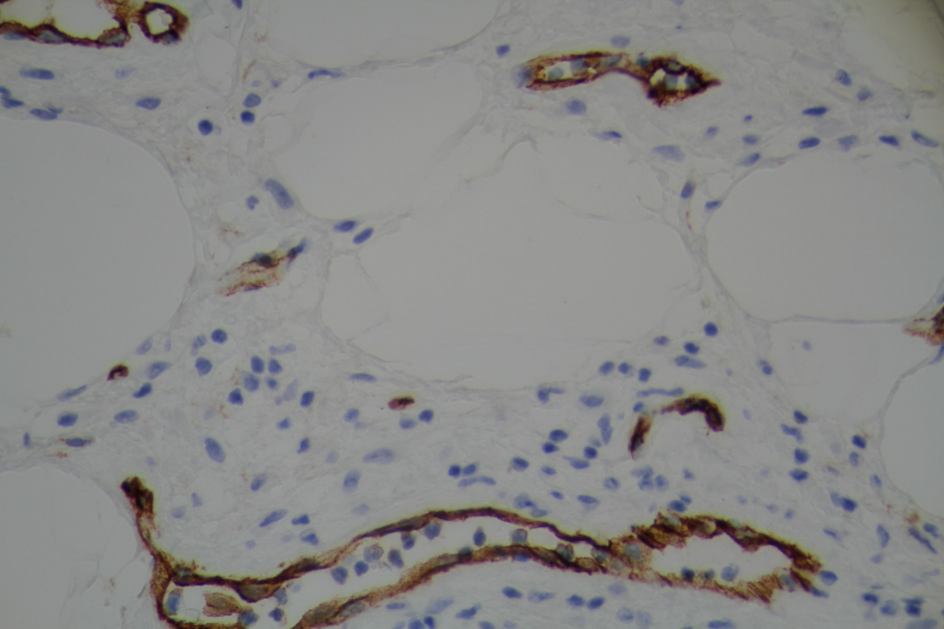
Immunohistochemical staining demonstrating expression of actin in smooth muscle cells and blood vessels.

**Figure 6:**
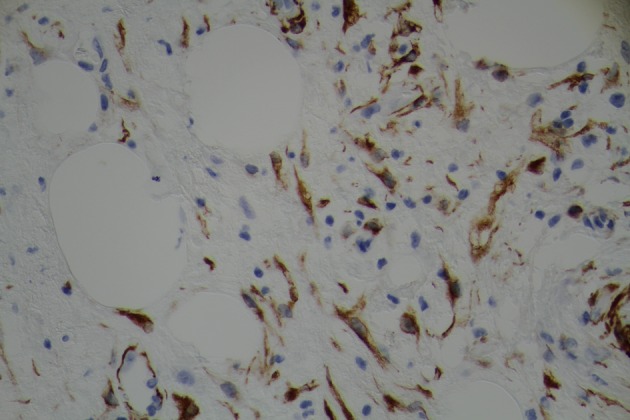
Immunohistochemical stain for CD34.

Surgical team was consulted and resection of gastric mass was planned. On laparoscopy, a mass was seen in the distal stomach which appeared to be intraluminal only. Through a small upper abdominal incision, anterior gastrostomy was done and the mass was delivered through the opening. Mass was entirely within the wall of stomach with overlying ulcer. A submucosal resection of the mass was done and sent for frozen section analysis. Intra operatively, it was consistent with intramural lipoma. Gross evaluation of the specimen revealed a homogeneous yellow-tan colored submucosal fatty mass measuring 6 × 3 × 3cm. There were no areas of hemorrhage or necrosis. Microscopic examination showed a circumscribed nodule composed primarily of mature adipose tissue with prominent vascularity (Fig. 3). There were scattered foci of smooth muscle proliferation which were desmin (Fig. 4) and smooth muscle actin positive (Fig. 5). CD 117, human melanoma black (HMB)-45 were negative. CD34 highlighted the prominent vascularity (Fig. 6). No cytologic atypia, appreciable mitotic activity or necrosis was noted. The final pathological diagnosis was a benign gastric angiomyolipoma.

## Discussion

Angiomyolipoma (AML) is a relatively rare neoplasm, composed of variable proportions of adipose tissue, spindle and epithelioid smooth muscle-like cells and abnormal thick-walled blood vessels [1]. Though AMLs are considered to be benign tumors, there are sporadic cases of either recurrent or malignant AMLs reported in the literature [2]. These tumors are common in kidney and account for 0.3 to 3.0% of all renal masses. They can occur sporadically or as part of a tuberous sclerosis complex (TSC) where 60-80% of tuberous sclerosis have AMLs [6, 7]. In a single institution study of 156 AMLs arising from different organs reported by Yang et al, two most commonly involved organs were kidney and liver, comprising of > 90% of the cases [3]. Other involved sites, as sporadically reported in literature are oral cavity (hard palate, lips, parotid gland), nasal cavity, chest wall, ear, breast, heart, lung, spinal cord, spermatic cord, penis, vaginal wall, adrenal gland, anterior mediastinum, pancreas, uterus and gastrointestinal tract [2-4].

In the gastrointestinal tract, other than in liver, it was reported in duodenum, appendix, pancreas and stomach [3, 4]. Gastric angiomyolipoma (GAML) is very rare and was reported only twice in the literature. GAML is a type of submucosal tumor (SMT). SMTs include benign, potentially malignant, and malignant lesions. These are being increasingly recognized during routine endoscopies, with a reported frequency of 1 in every 100 to 300 gastroscopic examinations. In one of the studies, it is estimated that about 13% of gastrointestinal SMTs were malignant, with the highest risk of malignancy in the stomach [8]. Most common gastric SMTs are leiomyomas followed by GIST. Others include lipoma, hemangioma, glomus tumor, lymphangioma.

EUS is the choice of investigation for the evaluation of SMTs [9, 10]. Based on EUS findings, further investigations and therapeutic options include mucosal resection and fine needle aspiration or surgery can be sought. Suspicious malignant findings on EUS includes a large lesion of > 3 - 5 cm of size, a lesion with irregular margins, cystic spaces, heterogenous echos [10]. Normal gastric wall can be distinguished into five distinct layers namely mucosal layer, muscularis mucosa, submucosal layer, muscularis proper layer and serosa. EUS helps in the identification of the layer of origin which further helps in differential diagnosis of the gastric SMTs. Usually myogenic tumours like GISTs arise from muscularis mucosa and muscularis proper layer where as lipomas, fibromas, cysts arise from submucosal layer [10]. Modalities of treatment of SMTs include endoscopic submucosal resection, laparoscopic resection or traditional laparotomy and excision of the lesion. Endoscopic submucosal resection is feasible in small lesions (< 3 cm) that are limited to second or third layer and cannot be done if the lesion involves the whole gastric wall. Laparoscopic procedures include wedge resection or partial gastrectomy [11].

The pathological diagnosis of AML depends on demonstrating three cell lines namely blood vessels, epithelioid or spindle smooth muscle, and mature adipose tissue. The immunohistochemistry profile of AML is very variable. The monoclonal antibody which was previously raised against melanoma cell line, HMB-45 positivity is considered to be the most specific and characteristic feature of AML [12, 13]. HMB-45 antigen can actually help in differentiating renal AML from other renal tumors [12]. Other histochemical markers used in diagnosis of AML include smooth muscle actin (SMA), muscle-specific actin (MSA), vimentin, desmin, S-100 protein, CD34, CD117, cytokeratins [12-14]. In a recent large series of hepatic AML by Chang et al, the expression of HMB-45, SMA, and CD34 was 100%, 91.2% and 75.3% respectively. In another study, HMB-45, CD117 and SMA were expressed in 100%, 100% and 94% of the cases respectively [14]. The only case of gastric AML in literature with reported immunohistochemistry was HMB-45 positive [4]. Interestingly, in our case, the tumor expressed desmin, SMA (which indicates smooth muscle proliferation), CD34 (which highlights prominent vascularity) and was negative for HMB-45 and CD117.

In the first reported case by Helwig et al, patient presented with upper gastrointestinal bleed which showed 2 × 3 cm mass with central ulceration on endoscopy. The second case by Aggarwal et al, was a 40-year-old female who presented with vague abdominal pain, intermittent melena, hematemesis and found to have a 5 × 4cm polypoidal mass along the greater curvature of stomach. In the second case, tumor was positive for HMB-45 antigen and other immunohistochemical stain patterns were not reported. Laparotomy with excision of tumor was done in the first case where as laparoscopic wedge resection was performed in the second case.

### Conclusion

Gastric angiomyolipoma (GAML) is a tumor with distinct histopathological pattern which can be a very rare cause of upper gastrointestinal bleeding. Continued reporting of these cases is important to understand clinicopathological characteristics and malignant potential. So far, GAML is a benign tumor and complete resection is curative.
